# Neuroimaging predictors of cognitive resilience in the presence of Alzheimer’s disease pathology

**DOI:** 10.1002/alz.092917

**Published:** 2025-01-09

**Authors:** McKenna E. Williams, Christine Fennema‐Notestine, Tyler R. Bell, Shu‐Ju Lin, Stephen J. Glatt, William S. Kremen, Jeremy A. Elman

**Affiliations:** ^1^ SDSU/UC San Diego Joint Doctoral Program in Clinical Psychology, San Diego, CA USA; ^2^ University of California, San Diego, La Jolla, CA USA; ^3^ SUNY Upstate Medical University, Syracuse, NY USA

## Abstract

**Background:**

Structural brain measures derived from magnetic resonance imaging (MRI) are commonly used to quantify Alzheimer’s disease (AD) progression, though their relationships with cognitive performance are highly heterogeneous. Some individuals demonstrate greater cognitive resilience—the ability to maintain cognitive performance despite adverse brain‐related changes—through as yet unknown mechanisms. We examined whether cortical thickness in specific regions confers resilience cross‐sectionally and/or longitudinally by moderating the relationships between established markers of AD risk and cognitive performance in amyloid‐positive adults.

**Method:**

Amyloid‐positive participants from the Alzheimer’s Disease Neuroimaging Initiative (ADNI) GO/2/3 cohorts with relevant imaging data were included (*n*=160, observations=473). Risk markers included a validated cortical thickness/hippocampal volume AD risk signature (weighted sum of cortical thickness from 7 regions plus hippocampal volume) and cerebrospinal fluid phosphorylated tau (p‐tau). Linear mixed effects models tested whether region‐specific cortical thickness moderated relationships between these markers of AD risk and memory or executive functioning.

**Result:**

Cross‐sectionally, thicker cortex in a composite of 8 regions of interest (ROIs) minimized the negative impact of thinner cortex in AD risk signature regions on executive function performance. Higher thickness in a resilience composite of these 8 ROIs at baseline was associated with less decline in memory over time (*p*=0.007), independent of effects of other markers of AD risk. Moreover, higher baseline resilience composite scores weakened the negative longitudinal effects of p‐tau (*p*=0.014), independent of the longitudinal effects of AD risk signatures and age.

**Conclusion:**

We identified 8 cortical regions that appear to confer cognitive resilience cross‐sectionally and longitudinally in the face of established indicators of AD pathology. Among amyloid‐positive individuals, thicker cortex in these resilience regions was cross‐sectionally associated with better executive function despite thinner cortex across established AD risk signature regions. Despite any cross‐sectional association with memory, thicker cortex in resilience regions predicted slower decline in memory over time and weakened the negative effects of p‐tau on memory over time. Preserved executive functioning associated with cortical thickness in these resilience ROIs may enable compensation in subsequent memory performance over time. These findings provide insight into possible mechanisms of cognitive resilience and individual variability in cognitive trajectories associated with AD.

